# Hepatitis E Virus Antibodies in Patients with Chronic Liver Disease

**DOI:** 10.3201/eid1503.080740

**Published:** 2009-03

**Authors:** Muslim Atiq, Norah J. Shire, Anna Barrett, Susan D. Rouster, Kenneth E. Sherman, Mohamed T. Shata

**Affiliations:** University of Arkansas for Medical Sciences, Little Rock, Arkansas, USA (M. Atiq); University of Cincinnati College of Medicine, Cincinnati, Ohio, USA (N.J. Shire, A. Barrett, S.D. Rouster, K.E. Sherman, M.T. Shata)

**Keywords:** Hepatitis E, chronic liver disease, seroprevalence, dispatch

## Abstract

In the United States, the seroprevalence rate for hepatitis E virus (HEV) is ≈20%. This study examined HEV seroprevalence in persons with and without chronic liver disease. Our data indicate that HEV seropositivity is high in patients with chronic liver disease and that HEV seroprevalence increases significantly with age.

The hepatitis E virus (HEV) is an enterically transmitted, hepatotropic, single-stranded RNA virus. It causes a self-limiting acute infection, but the infection does not develop into chronic disease in immunocompetent persons. Although symptoms are generally mild, they may be severe in certain cases, especially in pregnant women (*1*). HEV is endemic in regions where the water supply may be contaminated with animal waste, such as in Central Asia, the Middle East, and parts of South America and Africa. Large waterborne epidemics have occurred in refugee camps in Somalia (*2*) and Sudan (*3*), causing sickness and death. In regions where HEV is endemic, seroprevalence and illness rates for hepatitis E infection vary from 40%–90% (*4*,*5*). In the United States, seroprevalence studies detected antibodies against HEV (anti-HEV) in >21% of blood donors (*6*). Despite this indication of its presence in the United States, HEV infection is rarely diagnosed in this country except in travelers to HEV-endemic areas (*7*).

Recent reports have demonstrated that zoonotic (swine) reservoirs may exist in non–disease-endemic regions (*8*) and that unrecognized HEV may be the source of cryptogenic hepatitis in certain cases (*9*). Data concerning immunocompromised patients are scarce; however, higher seroprevalence of HEV has been reported in HIV-positive persons than in the general population. Additionally, in patients with compromised immune systems, symptoms of HEV infection and viremia may be prolonged (*10*,*11*). We conducted a study to determine the seroprevalence of anti-HEV in persons with and without chronic liver disease to uncover potential associations between clinical markers of liver disease and HEV seroprevalence and to identify risk factors associated with HEV positivity.

## The Study

We performed an observational cross-sectional analysis of de-identified serum samples and data from patients with chronic liver disease who were monitored at the University of Cincinnati, Cincinnati, Ohio. Data and samples were collected during 1995–2006. Healthy donor samples were obtained from controls (generally healthcare and laboratory personnel) who had no evidence of liver disease. Limited demographic, clinical, and laboratory data were collected from medical records and existing databases for patients with chronic liver disease and for controls. Tests for anti-HEV immunoglobulin (Ig) G and IgM were performed by ELISA (MP Biomedicals Asia Pacific Pte Ltd, Singapore; formerly Genelabs Diagnostic Pte Ltd) according to the manufacturer’s instructions. Assay validity was evaluated according to the manufacturer’s specifications. Demographic, clinical, and laboratory values were reported as mean ± SD for normally distributed variables and as median (range) for nonnormal continuous variables. Between-group comparisons were assessed by analysis of variance with the Sidak adjustment for multiple comparisons, the Kruskal-Wallis test, the χ^2^ test, or Fisher exact test, as appropriate to the data distribution. Logistic regression was used to determine predictors of IgG positivity. A 2-tailed p value <0.01 was considered significant in all cases.

**Figure 1:**
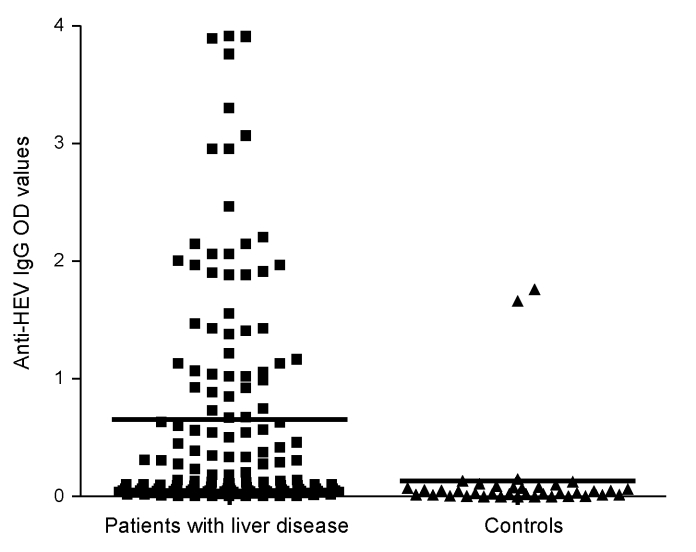
Relationship between anti-hepatitis E virus (HEV) immunoglobulin (Ig) G and chronic liver disease. Scattered plot of the optical density (OD) of anti-HEV IgG for both liver disease patients and control groups is shown; each point represents a subject. Means (0.63 and 0.13 for patients with chronic liver disease and for control group, respectively) are plotted as horizontal lines.

**Figure 2:**
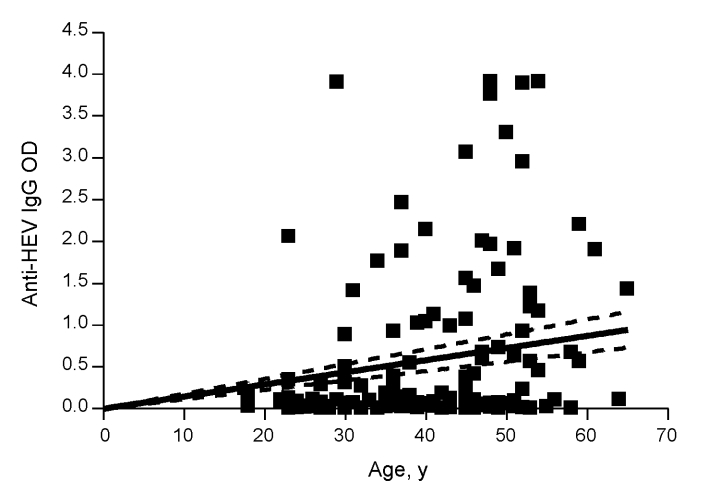
Correlation between the optical density (OD) of anti-hepatitis E virus (HEV) immunoglobulin (Ig) G and the age of persons. OD values of the anti-HEV IgG were plotted against the age of the enrolled persons. Correlation between age and OD value is significant (p <0.0001, r = 0.305).

A total of 167 persons with a mean age of 39.6 years (+10.9 years) were evaluated. Of these, 129 had chronic hepatitis (46 [35.7%] of whom were co-infected with HIV), and 38 were healthy controls. Demographic and laboratory characteristics are displayed in Table 1. The most common causes of chronic liver disease were hepatitis C virus (HCV) infection, present in 59 (45.7%) of the 129 persons, and HCV/HIV co-infection, present in another 39 (30.2%). Other causes of liver disease included hepatitis B virus (HBV) infection (7.8%), HBV/HIV co-infection (6.2%), autoimmune hepatitis (3.1%), and idiopathic hepatitis (6.9%). Of those with liver disease, 89.9% had viral infections and 30.2% had alanine aminotransferase (ALT) levels >2× the upper limit of the reference range. Eighty-four patients had risk factors assessed, and 68 of those had a known risk factor for viral hepatitis. These risk factors included being an injection drug user or man who has sex with men, using cocaine nasally, having had a blood transfusion, and having hemophilia. Forty-nine (29.3%) of the 167 persons evaluated tested positive for HEV IgG antibody, and most of these persons (45/49, 91.8%) had chronic liver disease. None were positive for anti-HEV IgM, a marker for acute HEV infection. Optical densities (ODs) for anti-HEV IgG differed significantly (p<0.001); the group with liver disease had greater values than the controls (mean 0.63 ± 0.96 and 0.13 ± 0.38, respectively; median 0.12 and 0.03, respectively), as shown in Figure 1. No relationship was shown between anti-HEV IgG ODs and the cause of liver disease. Because the odds of HEV seropositivity increase with age, correlation between age and ODs was examined; ODs increased significantly with age (p<0.001, Spearman rank correlation r = 0.305) (Figure 2).

**Table 1:** Characteristics of 167 persons evaluated in study of association between HEV and chronic liver disease*[REMOVED HYPERLINK FIELD]

Characteristic	No. (%) persons
Gender	
M	100 (59.9)
F	67 (40.1)
History of chronic liver disease	
Yes	129 (77.2)
No	38 (22.8)
HIV co-infection	
Yes	46 (27.6)
No	121 (72.4)
HEV IgG	
Positive	49 (29.3)
Negative	118 (70.7)

*HEV, hepatitis E virus; IgG, immunoglobulin G.

To determine characteristics associated with testing positive for HEV, univariable logistic regression was conducted. Predictors of significance included age (p = 0.002), male gender (p = 0.009), liver disease (p = 0.001), and HIV co-infection (p = 0.04). A multivariable regression model was then developed with age and liver disease and age by liver disease interaction as predictors. The interaction was not significant (p = 0.97) and, therefore, was removed from the model. The final model showed that the chance of being HEV positive increases as age increases (adjusted odds ratio [AOR] 1.05, p = 0.002). Also, probability of HEV positivity is significantly greater if other liver disease is present (AOR 7.78, p<0.001) (Table 2). Male gender was significantly associated with HEV positivity (AOR 2.07, p = 0.009), but no associations were found between HEV positivity and race/ethnicity, viral versus nonviral liver disease, ALT level, or HIV seropositivity.

**Table 2:** Hepatitis E virus immunoglobulin G multivariable logistic regression results

Risk factor	Adjusted odds ratio	SEM	p value	95% Confidence interval
Age	1.05	0.02	0.002	1.02–1.09
Male gender	2.07	0.07	0.009	1.17–3.66
History of chronic liver disease	7.78	3.25	0.001	3.43–17.64

## Conclusions

HEV in the United States and other industrialized nations is not thought to present a substantial risk of illness and death; however, recent data suggest that in these regions, HEV may be a cause of cryptogenic hepatitis. In Japan, HEV RNA has been detected in blood donors with elevated liver enzymes (*12*). A recent report from France found that acute HEV in persons who had not traveled to HEV-endemic regions may cause fulminant hepatitis, especially in those with active alcohol use and other chronic liver disease (*13*). Despite these associations and evidence of decompensation in patients with cirrhosis from preexisting liver disease in developing countries (*14*,*15*), whether HEV causes fulminant hepatitis among those with chronic liver disease who live in the United States is unknown.

Our study has limitations that should be noted. First, the intent of this analysis was to determine whether an association between HEV and chronic liver disease was detectable. We therefore used an observational study design. The results of the study should be carefully interpreted to avoid assumptions of a cause-effect relationship, as this study was a cross-sectional survey. Second, 2 discrete populations were included. Differences in sample collection dates, geographic location, and other variables could affect results, although no evidence of any confounding effect was detected. Third, as is the case in many retrospective analyses, we were unable to collect all risk factors and characteristics of persons in the study. Underreporting of injection drug users, for example, would undermine power for detection of an association between drug use and HEV seropositivity. However, collecting data on behavior risk factors for viral hepatitis poses a challenge even in prospective studies, and our results are consistent with expectations.

Whether HEV superinfection mediates other liver disease in persons living in the United States can be determined only by prospective cohort studies. Such follow-up is warranted to determine the role of HEV superinfection as a participating factor in liver diseases.
